# Development of Redox-Active Lyotropic Lipid Cubic Phases for Biosensing Platforms

**DOI:** 10.1021/acs.langmuir.3c02307

**Published:** 2023-12-19

**Authors:** Wanli Liu, Simon E. Lewis, Mirella di Lorenzo, Adam M. Squires

**Affiliations:** † Department of Chemistry, University of Bath, Bath BA2 7AY, U.K.; ‡ Department of Chemical Engineering, University of Bath, Bath BA2 7AY, U.K.

## Abstract

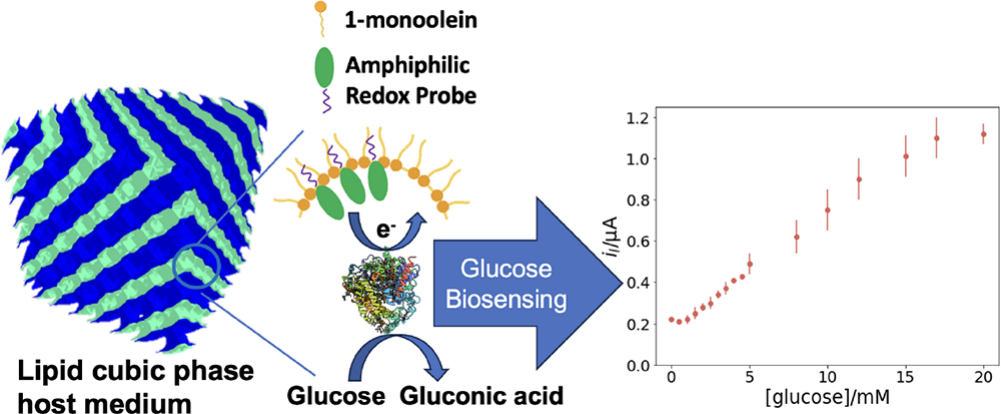

Enzyme-based electrochemical biosensors play an important role in point-of-care diagnostics for personalized medicine. For such devices, lipid cubic phases (LCP) represent an attractive method to immobilize enzymes onto conductive surfaces with no need for chemical linking. However, research has been held back by the lack of effective strategies to stably co-immobilize enzymes with a redox shuttle that enhances the electrical connection between the enzyme redox center and the electrode. In this study, we show that a monoolein (MO) LCP system doped with an amphiphilic redox mediator (ferrocenylmethyl)dodecyldimethylammonium bromide (Fc12) can be used for enzyme immobilization to generate an effective biosensing platform. Small-angle X-ray scattering (SAXS) showed that MO LCP can incorporate Fc12 while maintaining the Pn3m symmetry morphology. Cyclic voltammograms of Fc12/MO showed quasi-reversible behavior, which implied that Fc12 was able to freely diffuse in the lipid membrane of LCP with a diffusion coefficient of 1.9 **±** 0.2 **×** 10^–8^ cm^2^ s^–1^ at room temperature. Glucose oxidase (GOx) was then chosen as a model enzyme and incorporated into 0.2%Fc12/MO to evaluate the activity of the platform. GOx hosted in 0.2%Fc12/MO followed Michaelis–Menten kinetics toward glucose with a *K*_M_ and *I*_max_ of 8.9 **±** 0.5 mM and 1.4 **±** 0.2 μA, respectively, and a linearity range of 2–17 mM glucose. Our results therefore demonstrate that GOx immobilized onto 0.2% Fc12/MO is a suitable platform for the electrochemical detection of glucose.

## Introduction

Electrochemical biosensors play a significant role in point-of-care diagnostics,^1,2^ with examples of portable and wearable devices able to monitor biomarkers of relevance in serial physiological fluids such as blood,^3^ saliva,^4^ sweat,^5^ and tears.^6^ Coupling with cost-effective miniaturized electronics and wireless communication allows the information gathered by such biosensors to be remotely transmitted via a mobile phone to a doctor^7^ minimizing delays and costs associated with sample collection and analysis in traditional clinical settings and therefore enhancing intervention and management of diseases.^8^

Enzymes allow the development of highly specific electrochemical biosensors.^9^ Nonetheless, the development of effective enzyme-immobilized electrodes still represents a major challenge in these devices^10−12^ Current approaches for enzyme-based electrode fabrication include chemical cross-linking via redox-active hydrogels^13,14^ and physiological entrapment in biomimetic media.^15^ Although both strategies have been well adopted, showing promising results, there are a few associated drawbacks that hinder practical applications. These include high costs, low product yield, complex and time-consuming fabrication protocols, and poor reproducibility.^16,17^

**Figure 1:**
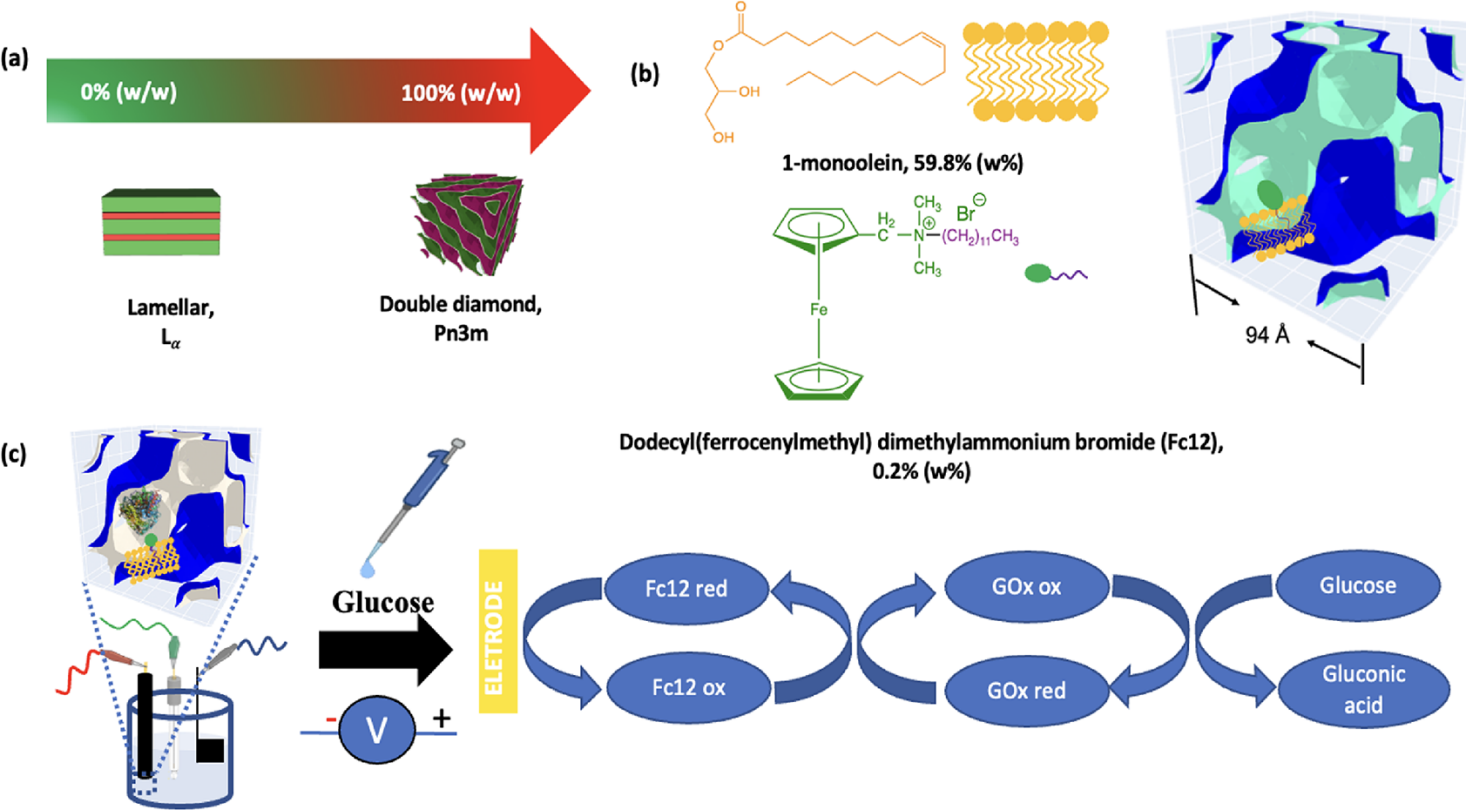
(a) Polymorphism of 1-monoolein in various hydration conditions at 25 °C;^24^ it stays in the 2D lamellar phase (L_α_) and changes its morphology into double-diamond (Pn3m) in bulk aqueous environments. (b) 1-Monoolein (MO) containing 0.2%Fc12 (w/w) forms lipid cubic phases (MO/Fc12/aqueous, 59.8/0.2/40, w/w/w) with Pn3m symmetry in excess aqueous condition, the blue and light-green regions represent lipid bilayers and aqueous channels, respectively. (c) Schematic experimental setup: an Au disk electrode coated with 0.2%Fc12/MO/glucose oxidase (GOx) lipid cubic phases is placed in a phosphate buffer, and concentrated glucose solution is added to the electrolyte dropwise and novel glucose sensing is stimulated by the potential sweeping bias.

Recent research has focused on the use of bicontinuous lipid cubic phases (LCP) as a method to immobilize enzymes for electrochemical devices.^18,19^ These ordered nanomaterials comprise 3D nanoscale water channel networks separated by a curved lipid bilayer^20^ and provide ideal scaffolds to host enzymes in an electrochemical device. The nanostructure can entrap enzymes while the water channel networks allow 3D diffusion/transport to/from the surrounding electrolyte and the electrode surface.^21^ In addition, the LCP nanostructure forms spontaneously when the lipid is mixed with aqueous solution (Figure 1a) and adheres to the electrode surface, forming a stable coating in the analyte solution, which is based on readily available plant-derived lipids. LCP therefore represents an attractive platform for the development of enzymatic biosensing technology. Several successful electrochemical systems have been recently reported in which LCP is used to encapsulate various types of enzymes including hydrophilic^21^ and membrane bound.^22,23^

However, the effective development of LCP-entrapped enzyme electrodes requires the use of a redox shuttle molecule that facilitates the electron transfer between the enzyme and the electrode. With this purpose, water-soluble redox shuttles have been so far employed in the electrolyte, such as hydroquinone,^25^ hexamine ruthenium,^26^ and ferrocene carboxylic acid.^26−28^ The feasibility of using the ferrocene-mediated enzyme electrode in biosensing was first demonstrated by Cass et al.^29^ While effective, the use of redox shuttles in the electrolyte is impractical for the development of biosensors to be used in real-world contexts. Barauskas et al.^30^ directly entrapped hydrophobic ferrocene derivatives within the LCP-forming lipid monoolein. However, the hydrophobic redox molecules likely located within the hydrocarbon part of the bilayer, not accessible to membrane proteins in the aqueous channels; higher levels induced a phase transition from the 3D LCP geometry to the inversed hexagonal phase (H_II_), which prevented the 3D diffusion in the LCP pockets within the nanostructure, thus affecting electrochemical performance.^31^

Nonferrocenium redox-active LCP systems were also reported. The phase behavior and electrochemical properties of ubiquinone-10/MO LCP were presented by Razumas et al.^32^ Ubiquionone-10 is a 1,4-benzoquinone that has a long side chain with 10 branched isoprenoid subunits. Subsequent studies indicated that ubiquinone-10 has a rather low solubility in LCP and tend to crystallize at >0.5 wt %, as well as promoting a phase transition of LCP→ H_II_.^33,34^ A similar behavior *in meso* was also reported for the structural analogue vitamin-K_1_ (phylloquinone) that has prenyl subunits. The Pn3m phase of monoolein LCP has low tolerance to vitamin-K_1_ and transforms into H_II_ when >1 wt % of vitamin-K_1_ is present *in meso*.^35,36^ Below the solubility threshold, both benzoquinone derivatives have been found to be electrochemically irreversible over the pH range of 6–8.^32,35^ The low solubility and inefficient electron transfer in LCP have restricted benzoquinone-LCP systems from further electrochemical developments.

In this study, we report for the first time the development of a redox-active LCP that incorporates the amphiphilic shuttle dodecyl(ferrocenylmethyl)dimethylammonium bromide (Fc12) within the cubic phase formed by the lipid monoolein (Figure 1b). The Fc12 molecule has a hydrophobic “tail” that is incorporated into the lipid bilayer of 1-monoolein (MO), and a redox-active polar “head” located within the aqueous water channels. The risk of leaching of the hydrophilic analogue is therefore prevented, and so is the risk of phase transition to H_II_ from the hydrophobic one, while redox activity in the headgroup allows electron transfer from the enzyme redox center to the electrode. The use of this redox-active LCP as a platform for the effective development of an electrochemical biosensor is assessed by considering glucose oxidase as the model enzyme (Figure 1c). The resulting probe is tested for glucose detection.

## Methods

### Materials

Glucose oxidase (E.C.1.1.3.4 from *Aspergillus niger*, Sigma), d-(+)-glucose (Sigma), sodium phosphate dibasic (Sigma), dodecyl(ferrocenylmethyl)dimethylammonium bromide (purity >97%, Tokyo Chemical Industry Co., Ltd.), and sodium hydroxide (99.5%, Schlau, Germany) were used as received. Monoolein (1-oleoyl-*rac*-glycerol) was purchased from Croda (Cithrol GMO HP-SO-LK, purity >96%). All solutions were prepared by using Milli-Q water (18.2 MΩ cm^–1^, Millipore, Bedford, Massachusetts, USA). Glucose solutions in 50 mM phosphate buffer solutions (pH = 7.0) were prepared at least 24 h before the experiments to equilibrate between α and ß anomers.^37^

### Preparation of Fc12-Doped Monoolein and Lipid Cubic Phase Paste

Monoolein (MO) was placed in a water bath and heated at 40 °C for 15 min. Fc12 and molten MO were weighed out according to the w/w ratios and added to a 2 mL Eppendorf tube. The matrix was quickly vortexed and placed in an ultrasonic water bath sonicator. The mixture of Fc12 and MO was then sonicated in a 40 °C water bath for 10 min; full dissolution of Fc12 in MO was examined by inspecting the clarity of the sample.

The solution of glucose oxidase (GOx) was prepared by dissolving 4–5 mg of GOx in 100 μL of 50 mM phosphate buffer (PB) in 2 mL Eppendorf tubes.

A small amount of Fc12/MO (∼60 mg) was pipetted and added to a 2 mL glass vial followed by addition of phosphate buffer with or without enzymes, at a 60/40 (w/w, lipid/aqueous) ratio. The matrix was vortexed and stirred at room temperature for 10 min to yield lipid cubic phases with and without the enzyme, leading to Fc12/MO/GOx and Fc12/MO, respectively. The “blank” lipid cubic phases containing no Fc12 or GOx were prepared following the above procedure, but the molten MO was mixed with an appropriate amount of phosphate buffer. The formation of lipid cubic phases was confirmed by macroscopic observation of the sample viscosity and clarity, and the phase identity was determined by SAXS experiments.

### Preparation of Lipid Cubic Phases for SAXS Experiments

Quartz capillaries (Capillary Tube Supplies Ltd., Q/1.5/OP/75/0.01) filled with LCP coatings and buffer solutions were prepared for SAXS studies as follows: approximately 100 μL of molten Fc12/MO was injected into a capillary, and excess lipid was drained out to give a thin layer of coating on the inner wall of ca. 10 mg (0.1–0.2 mm thickness). The capillary was subsequently filled with 100 μL of 50 mM PB buffer and sealed with heat shrinkers. Each capillary contained a lipid/aqueous matrix with approximately mass/volume ratio of 1/10 (w/v, lipid/aqueous). This gives an excess aqueous environment as in the electrochemical setup, for which the electrode with an LCP coating is dipped into the bulk electrolyte solution. The SAXS patterns of the LCP with immobilized enzymes were collected using an Anton Paar Multiple-Solid Sample Holder, which comprises two metal plates with 5 × 4 grids (11 × 11 mm for each grid) sandwiching the samples between two Kapton sheet windows. Appropriate amounts (ca. 10 mg each) of the LCP pastes were transferred into different cells of the sample holder, followed by topping with 10 μL of 50 mM PB solution.

The 2D SAXS patterns of the prepared samples were collected on a Dectris Eiger detector from an Anton Paar SAXS Point 2.0, using Cu source K_α_ radiation (λ = 1.54 Å), with a sample–detector distance of 572 mm and an acquisition time 10 min for each sample. The data reduction of the 2D to 1D radial profile was performed using Anton Paar SAXS analysis software by azimuthal integration of 330°.

### Electrode Modification

A Au disk electrode (PalmSens, ca. 2 mm in diameter) was polished with two grades of Al_2_O_3_ powders (0.3 and 0.1 μm) on wet polishing cloths, followed by sonicating in water for 5 min and then in ethanol for another 5 min. The Au electrode was activated in 0.5 M H_2_SO_4_ by sweeping the potential between 0.4 and 1.6 V versus Ag|AgCl (sat. KCl) for 10 min, and then rinsed with water and ethanol, and dried under N_2_ purge. Two pieces of Scotch tape were attached to either side of the circular electrode surface, leaving a cuboid void (*l* × *w* × *h*, 5 × 2 × 0.2 mm) in the middle, which was subsequently filled with a layer of LCP (60:40 w/w, lipid/aqueous as above). The excess LCP thin film was scraped off with a spatula. The weight of the LCP thin film was obtained by determining the difference between the bare and modified Au electrodes. The deposited thin film resulted to have a thickness of 0.2 mm and a weight of ca. 3–4 mg.

### Electrochemical Characterization of the Electrodes

Cyclic voltammetry was performed in a conventional three-electrode cell setup comprising an LCP-modified Au disk working electrode, a Ag|AgCl reference electrode (sat. KCl), and a Pt mesh counter electrode using an EmStat3+ PalmSens potentiostat. The potential was swept between 0.3 and 0.85 V to 0.85 versus Ag|AgCl (sat. KCl), with the following scan rates: 0.1, 0.05, 0.025, and 0.01 V s^–1^. The order of application of the different scan rates was randomized to alleviate any time effect. The background electrolyte 50 mM phosphate buffer solution (pH 7.0) was deoxygenated by bubbling argon for at least 30 min prior to experiments under Ar. The modified electrode was soaked in the electrolyte during the deaeration before each experiment.^27^

The electrocatalytic activity of Fc12/MO/GOx was investigated by cyclic voltammetry at 0.01 V s^–1^, in the absence and presence of glucose at concentrations within the range 0–20 mM. The activity toward glucose was further characterized by chronoamperometry at an applied potential of 0.85 V vs Ag|AgCl (sat. KCl) at increasing concertations of glucose, ranging from 0 to 20 mM.

## Results and Discussion

The effective incorporation of the Fc12 surfactant into the MO film was investigated to verify the presence of any change to the LCP morphology. The redox activity and diffusion within the MO film were also assessed.

**Figure 2:**
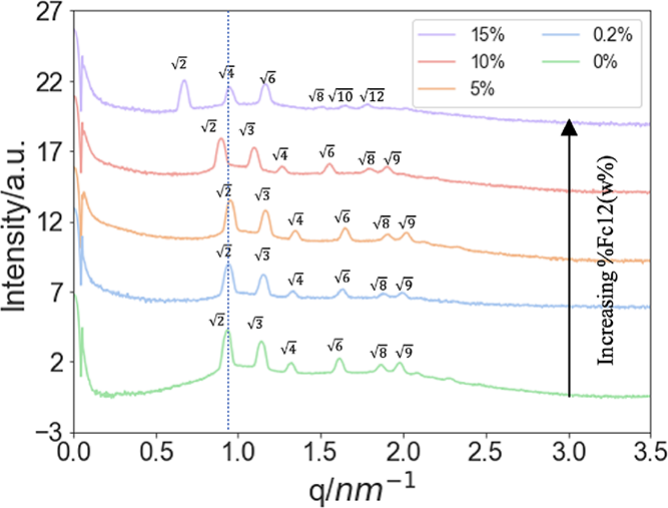
Effect of increasing the amount of Fc12 (w%) on the LCP morphology changes. SAXS patterns are obtained from Fc12/MO LCP in excess 50 mM PB aqueous environment (the inserted dashed line represents the first peak position of the 0%Fc12 sample).

SAXS was used to determine whether the presence of Fc12 and electrolyte buffer affects the LCP structure. Figure 2 shows SAXS patterns for various %Fc12/MO (wt %) compositions in the presence of excess 50 mM phosphate buffer, which is the electrolyte used in subsequent electrochemical experiments. With no or small fractions of Fc12 up to 5 wt %, the peak position ratios are consistent with a Pn3m symmetry LCP with lattice parameters of approximately 9.3–9.4 nm. The result is consistent with published data for MO in excess water.^38^ On increasing the Fc12 content, the lattice parameter increases to 9.8 nm for 10% Fc12 (as shown by a small shift of all peaks toward smaller angles), followed by a transition to an Im3m symmetry LCP. Fc12 therefore produces a less curved water–lipid interface, in which the space occupied by the polar head groups has become larger with increased incorporation of Fc12.^39^

These results confirm that Fc12 is embedded on the lipid bilayer of the LCP as an amphiphilic molecule, as desired. Hydrophilic molecules that completely dissolve in the LCP aqueous channels without interacting with the lipids do not significantly affect the nanostructure, while hydrophobic molecules that partition completely into the lipidic chain region produce more curved interfaces and transition into the H_II_ phase.^40^

**Figure 3:**
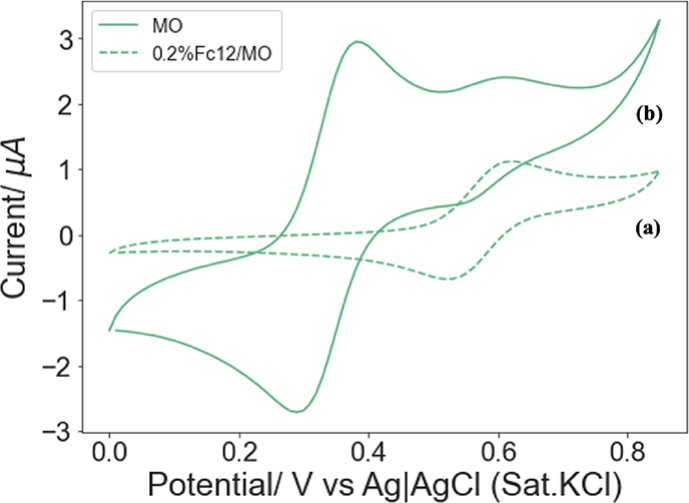
Cyclic voltammograms recorded at 0.1 V s^–1^ for (a) electrode modified with 0.2%Fc12/MO soaked in 50 mM PB solution (pH 7.0) and (b) electrode modified with MO-only cubic phases soaked in 1 mM Fc12 and 50 mM PB solution (pH = 7.0).

As complementary evidence that the Fc12 molecules are incorporated into the lipid membrane of the LCP, CV tests were also performed for an electrode modified with a coating of 0.2% Fc12/MO cubic phase (see Figure 3a). The relative curves collected show a classic reversible behavior.^41^ The formal potential *E*°_Fc12/Fc12_^+^ estimated from the mean value of the anodic and cathodic peak positions is 0.57 V. The Fc surfactant used in this work is soluble in water, with a critical micelle concentration of 0.5 mM in 0.2 M Li_2_SO_4_.^42^ On a clean gold electrode in the absence of LCP, Fc12 shows a single set of redox peaks with a formal potential of 0.41 V vs Ag|AgCl (0.43 V vs SCE), a significant lower potential than our value of 0.57 V for Fc12 within the MO LCP.^42,43^ The observed shift in the potential peak suggests a relative stabilization of the reduced form provided by the lipid membrane, in agreement with previous studies^27^^,^^28^ related to ferrocenecarboxylic acid in LCP, which reported a 0.03 V increase of formal potential for the hydrophilic redox molecule when LCP was present on the electrode surface. In addition, Opallo et al. found that the presence of torus-shaped cyclic oligosaccharides α-cyclodextrins in the bulk electrolyte led to an increased *E*°_app_ for Fc12.^43^ α-Cyclodextrins, which carry hydrophobic pores and a hydrophilic exterior, were able to stabilize the surfactant present in the aqueous solution by accommodating their hydrophobic chains into the hydrophobic interiors. However, the observed increase in *E*°_Fc12/Fc12_^+^ in LCP is 0.11 V, which is approximately four times greater than that of the hydrophilic ferrocenecarboxylic acid in LCP, which is 0.03 V.^28^ Therefore, the results provide further evidence, building on the SAXS data, that Fc12 is embedded in the lipidic region of the cubic phase and is not dissolved in the aqueous channels.

As a control, the LCP electrode was modified with a MO cubic phase only while Fc12 was dissolved in the electrolyte. In this case, two pairs of redox peaks were observed, as shown in Figure 3b, one at 0.38 V/0.30 V (*E*_pa_/*E*_pc_) and one at 0.61 V/0.55 V (*E*_pa_/*E*_pc_). These redox peaks suggested two different environments for Fc12. The more prominent peaks at 0.38 V/0.30 V likely correspond to Fc12 molecules that diffused through the aqueous channels to the electrode surface,^28^ while those at 0.61 V/0.55 V (*E*_pa_/*E*_pc_) correspond to those shown in Figure 3a, indicating that some Fc12 has become incorporated into the lipid bilayer. On a clean gold electrode without LCP, the redox surfactant only showed a single set of redox peaks with a formal potential of 0.41 V vs Ag|AgCl.^42^ The slightly higher potential relative to the potential of the lower potential redox couple we observed in Figure 3b, which we ascribe to molecules within the aqueous phase, could reflect the formation of micelles in their experiment.

It was noteworthy that the peaks at 0.61 V/0.55 V were relatively broader than those observed at 0.38 V/0.30 V. This result might be the result of a slower mass transport within the lipidic environment.^44^

### Diffusion Coefficient Measurement for Fc12 Immobilized in LCP

**Figure 4:**
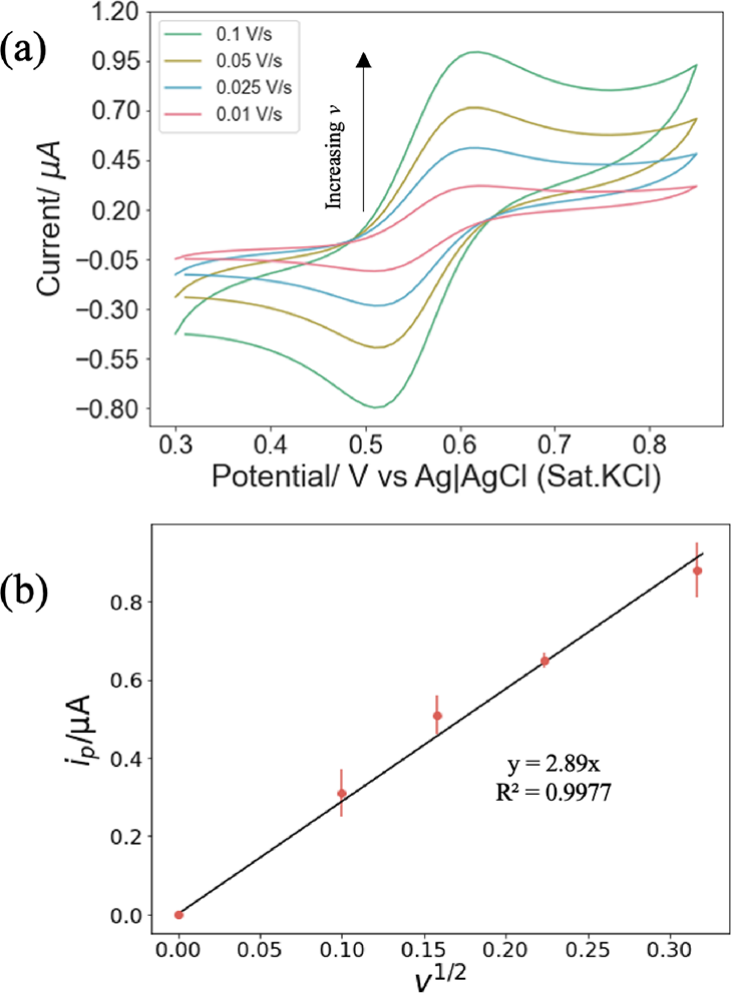
(a) Cyclic voltammograms recorded for the Au electrode modified with 0.2%Fc12/MO soaked in 50 mM PB solution (pH = 7.0) at varying scan rates: 0.1, 0.05, 0.025, and 0.01 V s^–1^. (b) Randles–Sevcik analysis: plotting anodic peak currents vs *v*^1/2^.

To measure the diffusion coefficient, CV scans at varying rates were recorded for 0.2%Fc12/MO-coated Au disk electrodes, as shown in Figure 4a. The diffusion coefficient of Fc12 hosted in the LCP was determined from the Randles–Sevcik equation assuming semi-infinite diffusion^44^
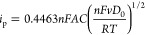
1where *i*_p_ is the maximum current (μA), *n* is the number of electrons involved in the redox reaction, *F* is the Faraday constant (96485.3321 s A mol^–1^), *A* is the electrode surface area (cm^2^), *C* is the concentration of the redox-active compound (mM), *D*_0_ is the diffusion coefficient (cm^2^ s^–1^), *v* is the voltage scanning rate (V s^–1^), *R* is the gas constant (8.3145 J mol^–1^ K^–1^), and *T* is the temperature (K).

The concentration of the redox probe Fc12 was estimated as per ref (45), where the LCP thin film was treated as a homogeneous medium. The 0.2% Fc12 (wt %) was completely incorporated into the lipid bilayer with an average concentration of 2.42 mM over the total volume of the thin film, which was calculated by assuming a density of 1 g cm^–3^. The active electrode was considered to be comparable to the geometric area of the electrode, 0.0314 cm^2^.

The anodic peak currents were obtained in triplicate measurements, and a linear relationship was obtained by plotting *i*_p_ vs *v*^1/2^ and forcing the line through the origin. The magnitudes of the anodic peak currents increased linearly as *v*^1/2^ increased (see Figure 4b) within the range of 0.3–0.85 V at low-voltage scan rates. These results illustrate that the mass transport of Fc12 is under diffusion control.^44^ The diffusion coefficient of Fc12 at 25 °C was estimated to be 1.9 ± 0.2 × 10^–8^ cm^2^ s^–1^.

Stability testing on repeated cycling showed that the system maintained 88% of its current after 50 cycles (ESI Figure 3). This confirms that the Fc12 molecules are not leaching significantly into the aqueous channels because this would lead to a greater drop in current. We can further rule out the possibility of Fc12 molecules being trapped within aqueous junctions of the lipid cubic phase (as with the GOx enzyme later in this paper) because our electrochemical data (Figure 4) demonstrates molecules that are free to diffuse through the lipid cubic phase with a consistent diffusion coefficient.

As reported in Table 1, the results align with the relevant literature values, although there are some discrepancies in the measured *D*_0_ values of ferrocene in the cubic matrix. There is no apparent trend between the entrapped molecule size and the diffusion coefficient, but the magnitudes of the diffusivities along lipid membranes were similar^30^ The *D*_0_ for different entrapped Fc derivatives varied a lot, because they could exhibit different aggregation form in the lipid membrane. For instance, ferrocene could have a similar *D*_0_ to an Fc derivative whose size is three times greater. Goss and Majda^46^ reported the *D*_0_ value of 2.7 × 10^–8^ cm^2^ s^–1^ for a ferrocene derivative Fc18 (FcCH_2_N^+^(CH_3_)_2_(CH_2_)_17_CH_3_), in the planar bilayer made of octadecyl trichlorosilane. Thus, the *D*_0_ determined by us fits the literature results well, given the fact that the redox-active compound is traveling through highly curved lipid/water interfaces.

**Table 1:** Diffusion Coefficient of Fc Derivatives in Varying Lipidic Environments

**compound name**	***D***_**0**_ **× 10**^**–8**^[cm^2^ s^–1^]	**diffusion medium**	**ref**
ferrocene	11	LCP, curved bilayer	(30)
ferrocene	2.7	LCP, curved bilayer	(45)
Fc18	2.7	planar lipid/water interface	(46)
Fc12	1.9	LCP, curved lipid/water interface	this work

### GOx Entrapment into the Redox-Active LCP Thin Films

The GOx enzyme was tested as a model enzyme and entrapped within the Fc12/MO mesophase. GOx was selected because it is low cost, easily accessible, robust, and highly specific toward glucose oxidation.^47−49^ It is one of the most ubiquitous enzyme candidates used for electrode fabrications to build blood sugar level diagnostic devices.^50^

**Figure 5:**
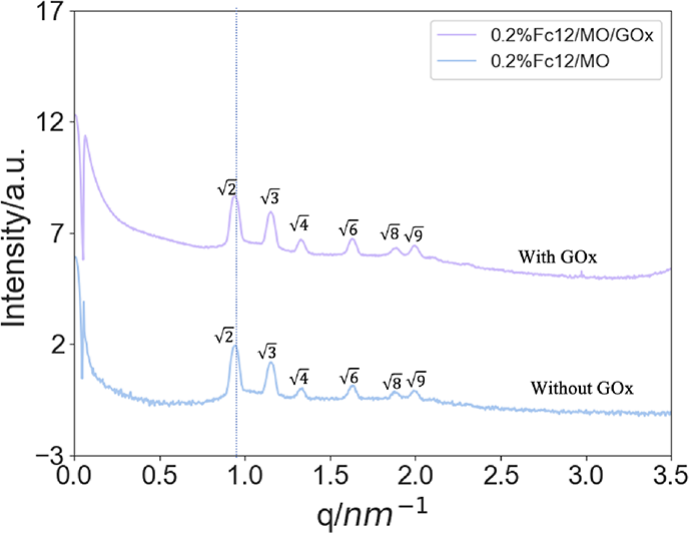
SAXS patterns of two different composition MO cubic phases produced by mixing melted 0.2%Fc12/MO with either (a) 50 mg mL^–1^ GOx and 50 mM PB (pH 7.0) solution or (b) blank 50 mm PB solution at 60/40 (w/w, lipid/aqueous). Samples were placed in an excess aqueous environment during the experiment (the inserted dash line represents the first peak position of 0.2%Fc12/MO).

Upon enzyme immobilization, SAXS analysis confirmed that the 0.2%Fc12/MO/GOx system maintained its cubic phase structure in the presence of the enzyme (Figure 5). The fact that the lattice parameter was unchanged implies that the enzyme is not incorporated into the bilayer but entrapped in the aqueous channels, as expected for this soluble enzyme.^21^ Two mechanisms have been suggested for entrapment of GOx within MO LCPs. The first is electrostatic^51^ whereby ionic interactions between GOx and the headgroup of monoolein enable the entrapment to take place. GOx has an isoelectric point (pI) of 4.2,^52^ which is lower than the pH of the buffer solution (pH 7). Therefore, net charges surrounding the enzyme molecules are formed and electrostatic repulsions between biomolecules are developed. The headgroup of the monoolein, which comprises two hydroxy groups of the glycerol moiety, starts to interact with the enzymes, shielding the net charges and entrapping them inside the water channel of the cubic phase. The second is steric:^53^ the radius of the water channels within the MO LCP is approximately 2.1 nm, as in MO in the absence of Fc12;^39^ Fc12 does not significantly affect the lattice parameter. This is slightly smaller than the size of the GOx, whose radius of gyration is estimated as 2.5 nm^54^ but which may be accommodated when trapped within the additional space of the junctions.^55^ The activity of GOx has been shown to be retained, alongside enhanced thermal stability upon the *in meso* entrapment.^56^

The Fc12/MO/GOx bioelectrode was subsequently tested for glucose sensing.
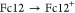
2
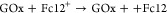
3
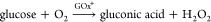
4

**Figure 6:**
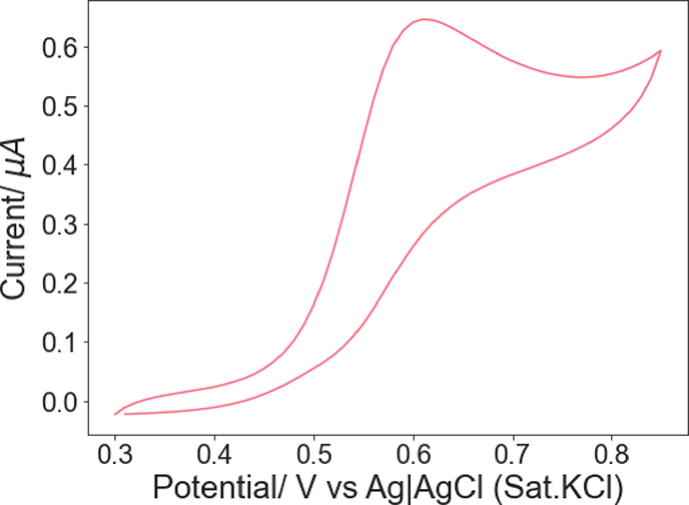
Cyclic voltammogram recorded for 0.2%Fc12/MO hosting 50 mg mL^–1^ GOx at 0.01 V s^–1^ in 5 mM glucose, 50 mM PB (pH = 7.0).

As shown in Figure 6, a marked anodic peak is observed related to the formation of Fc12^+^ (eq 2). The cathodic peak in the reversed scan is instead negligible, suggesting low reconversion of Fc12^+^, since most of the Fc12^+^ had reacted with the reduced form of GOx (eq 3);^41,57^ Fc12^+^, the product of the electrode reaction, participated in the catalytic reaction to regenerate the starting material Fc12 (eq 4). The reoxidized GOx enzyme can then carry out further cycles of glucose oxidation (eq 3).

**Figure 7:**
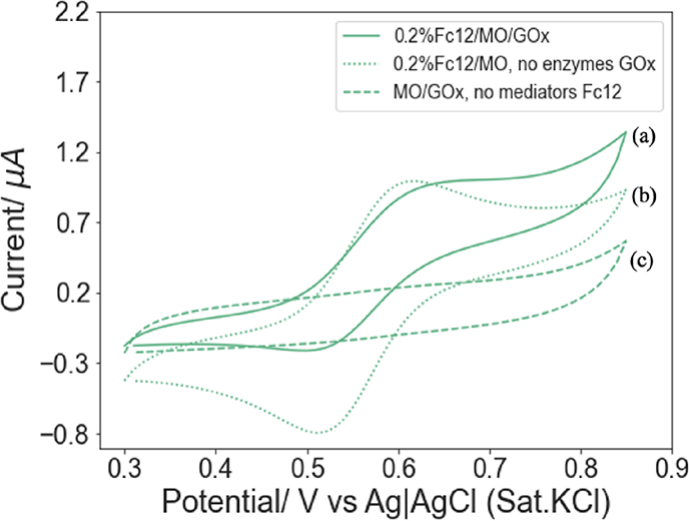
Cyclic voltammograms recorded for Au electrodes modified with different types of LCP thin films: (a) hosting both redox shuttle 0.2%Fc12 and enzyme 50 mg mL^–1^ GOx, (b) 0.2%Fc12/MO only, and (c) MO/GOx only at 0.1 V s^–1^ in 5 mM glucose, 50 mM PB (pH = 7.0).

Control experiments without GOx, and without the Fc12 shuttle, confirm the bioelectrocatalytic process and rule out other potential reactions that may produce the observed results. As shown in Figure 7c, in the absence of the Fc12 redox shuttle, no transient faradaic current was observed from the MO/GOx-modified electrode in a 5 mM glucose solution, suggesting that the glucose cannot be electrochemically oxidized directly, nor can an electrocatalytic enzymatic glucose oxidation reaction occur without the Fc12 shuttle, under the potential range used. Furthermore, in the absence of GOx, the LCP film containing solely 0.2%Fc12 exhibited a quasi-reversible behavior in a 5 mM glucose solution (see Figure 7b) and the ratio of anodic and cathodic peaks *i*_pa_/*i*_pc_ was 1. This result suggests that no Fc12 was captured by glucose for additional electrochemical interactions. For comparisons, Figure 7a shows the voltammogram of an LCP thin film hosting both 0.2%Fc12 and GOx under the same conditions, showing the characteristic electrocatalytic behavior, which is different from the controls.

**Figure 8:**
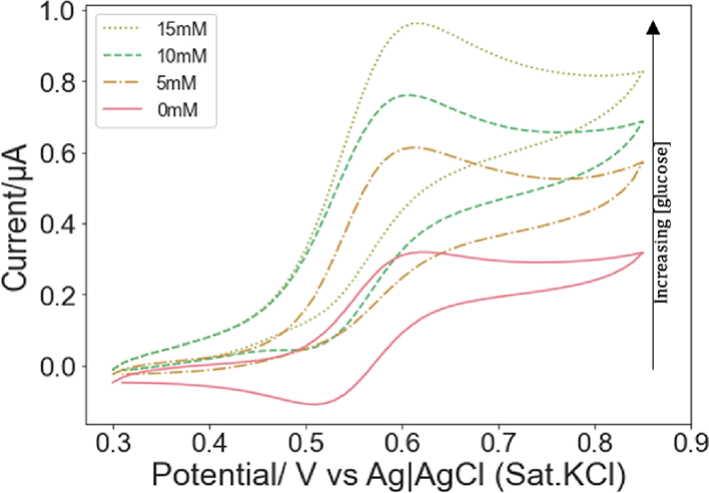
Cyclic voltammograms recorded for 0.2%Fc12/MO hosting 50 mg mL^–1^ GOx in a series of PB solutions (pH = 7.0) with varying glucose concentrations: 0, 5, 10, and 15 mM at 0.01 V s^–1^.

Cyclic voltammograms obtained with Fc12/MO/GOx exposed to different glucose concentrations are displayed in Figure 8. As shown, as the concentration of glucose increased, the anodic peak current also increased.^27^ The concentration of reduced Fc12 near the electrode built up and led to increasing anodic peak currents as the glucose content in the electrolyte increased.

**Figure 9:**
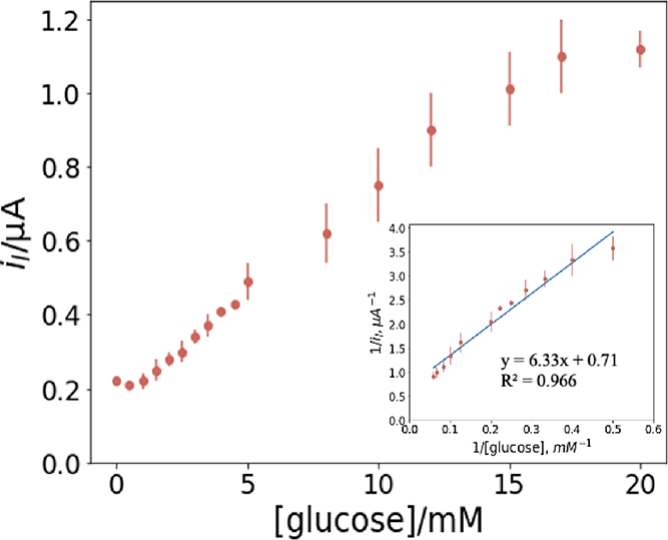
Limiting currents arise from glucose oxidation of the 0.2%Fc12/MO entrapping 50 mg mL^–1^ GOx system in different glucose PB solutions (pH 7.0), ranging from 0 to 20 mM; inset: Lineweaver–Burk plot for the amperometric signals obtained from glucose oxidation between 2 and 17 mM.

Chronoamperometry tests at the applied potential of 0.85 V were carried out to assess the kinetic parameters of immobilized GOx, and Figure 9 shows the limiting current obtained at various glucose concentrations. Below a glucose concentration of 2 mM, no detectable amperometric signal was observed. At higher concentrations, the amperometric signal increased linearly up to the value of 17 mM, where a plateau was observed. Therefore, the dynamic range of Fc12/MO/GOx was 2–17 mM, with a sensitivity of 1.8 μA mM^–1^ cm^–2^. This detection range covers the blood sugar level of diabetes patients, 6–8 mM.^58^

Enzyme kinetics analysis of the liquid crystalline supported GOx was performed by plotting the Lineweaver–Burk graph (see the inset of Figure 9):
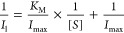
5where *I*_l_ is the maximum current (μA) at a certain glucose concertation, *K*_M_ is the Michaelis–Menten constant (mM), which accounts for the enzyme affinity, *I*_max_ is the maximum current (μA) generated from the reaction, and [*S*] is the substrate concentration (mM).^59^

The *K*_M_ and *I*_max_ of the immobilized enzyme were determined to be 8.9 ± 0.5 and 1.4 ± 0.2 μA, respectively. It is noteworthy that the bioelectrocatalyst has a relatively low *K*_M_ value for glucose, which is only 0.9 mM off the highest blood sugar level of diabetes patients.^58^ These results are comparable with other glucose sensors in LCP systems,^27^ with the advantage that the redox probe is embedded into the electrode and does not easily leach out.

The use of a redox molecule is key when enzymes such as GOx are used, which have the redox center deeply buried. Heller et al. pioneered the utilization of an Os redox polymer to facilitate electron transfer. The reported polymer network can either physically entrap^60^ or cross-link^61^ GOx on the electrode surface. However, the synthesis of the polymer Os(bpy)PVI involves harsh reaction conditions and it is time-consuming (>72 h).^62,63^ One alternative approach is tethering soluble redox probes to the enzyme backbone through flexible linkers, but it has similar issues regarding the usages of nongreen reagents.^64^ Nevertheless, the approach that is limited by the narrow spectrum of enzymes can be feasibly modified.^65^ Prior studies have provided insights into methodologies of electrical wiring enzymes to the electrodes but do not suggest practical routes to biosensing platforms that are low-cost, efficient, sustainable, and easy to prepare. The use of a redox-active lyotropic phase would have resolved the problems. Although the detection performance of the system we report here is less sensitive, with a narrower detection window than pre-existing systems using nanostructured semiconductors^66^ and other conductive materials,^67^ its performance easily satisfies the requirements for blood glucose sensing.^58^ It has a much simpler manufacturing process, leading to greater cost-effectiveness; the viscous matrices can be easily applied onto transducer surfaces and function well at room temperature. These properties suggest that the redox-active lyotropic crystal system holds considerable promise for low-cost sensor technology.

In the Fc12/MO/GOx electrode, the host medium MO is readily available without further chemical modifications, which enables practical applications. The immobilization protocol, based on GOx entrapment within the 3D nanostructure, is efficient and highly reproducible. Finally, the redox probe Fc12 is confined within the LCP matrix and self-assembles into the lipid/aqueous interface upon enzyme immobilization. Therefore, the results obtained show that the redox-active LCP method is compelling and promising in developing biosensing wearable healthcare electronics. The approach could be extended to other surfactants such as *N*-cetyl-*N′*-methyl viologen,^68^ which may be more accessible and would offer a range of different redox potentials to match the different enzymes.

The Fc12/MO system could also be applied to host other enzymes. The requirements for the potential candidates are first that there be a mechanism for entrapment of enzymes into the cubic phase imposing lower and upper physical size limits for soluble proteins. These soluble proteins are then preferred in this system as the ferrocene units of the redox probe sit within the aqueous channel. Second, the redox potential of the enzyme should be more negative than that of the redox probe within the cubic phase to allow electrocatalysis to take place. There are a number of potential strategies to expand the range of applicable enzymes. Tuning the chemistry of the redox probe can change its redox potential. Redesigning the probe so that the redox-active group lies within the hydrophobic part may allow electron transfer to the hydrophobic groups within membrane-bound proteins. Finally, the addition of other lipid molecules can swell the LCP water channels to incorporate larger soluble proteins.^69^

### Stability Test of Fc12/MO/GOx Bioelectrodes

We also studied the stability of our system over 14 days. For the stability tests, an Au disk electrode coated with Fc12/MO/GOx was placed in a 6 mM glucose PB solution and the electrochemical response arising from glucose oxidations was measured. The chronoamperometry setting for establishing the calibration curve was adopted for the stability test. Moreover, the modified electrode was stored in a PB solution at room temperature when not measuring.

As shown in ESI Figure 5, it was found that more than 80% of relative activity was retained for the first 4 days and then the activity started to fall over time. At the end of the stability test on day 14, there was only 20% of the relative activity left. The exact reason was not clear, but we suggest that the redox probe activity decay might cause the overall activity of the system to gradually drop.

## Conclusions

In the present work, we have demonstrated the feasibility of using an MO LCP platform for glucose sending by doping it with amphiphilic surfactant Fc12 and immobilizing the enzyme GOx. SAXS analyses confirmed the successful incorporation of Fc12 into the lipid bilayer of the MO LCP, while voltammetric studies confirmed its electroactivity. GOx was subsequently immobilized, showing electroactivity of the resulting electrode toward glucose.

We therefore presented a redox-active lyotropic phase capable of hosting an enzyme for electrochemical biosensing applications. The use of an amphiphilic redox shuttle that inserts into the bilayer produces a matrix that can maintain its continuous structure, allowing 3D diffusion of the redox shuttle and aqueous components to and from the electrode surface, while preventing leaching of the shuttle into external solution. The material has advantages over traditional biosensing platforms in the ease of preparation and deposition onto the electrode surface, avoiding the need for chemical steps, and it has the potential to accommodate a wide range of enzymes for sensing and catalytic applications.

## Supporting Information Available

Schematic representation of the SAXS sample preparation procedure, 2D SAXS images for all Fc12/MO cubic phases, peak indexing and lattice parameter calculation for all Fc12/MO cubic phases, stability test on repeated cycling of the 0.2%Fc12/MO-modified electrode, and long-term stability test of Fc12/MO/GOx bioelectrodes (PDF)

## Abbreviations

MO: monoolein
Fc12: (ferrocenylmethyl)dodecyldimethylammonium bromide
LCP: lipid cubic phase
GOx: glucose oxidase
