# Supplementary material for: Development of Redox-Active Lyotropic Lipid Cubic Phases for Biosensing Platforms
Source: Langmuir. 2023 Dec 19;40(1):170–8. doi: 10.1021/acs.langmuir.3c02307 (PMC10786026; doi:10.1021/acs.langmuir.3c02307)
Supplement: Supplementary file 1 — la3c02307_si_001.pdf [file la3c02307_si_001.pdf]

Supporting information

DEVELOPMENT OF REDOX ACTIVE LYOTROPIC LIPID CUBIC PHASES FOR BIOSENSING PLATFORMS

Wanli Liu[a], Simon E. Lewis[a], Mirella Di Lorenzo[b], Adam M. Squires[a]*

[a] Department of Chemistry, University of Bath, Bath, BA2 7AY, UK

[b] Department of Chemical engineering, University of Bath, Bath, BA2 7AY, UK

Table of Contents

ESI 1. Capillary sample preparation protocol for SAXS analysis S2

ESI 2. 2D SAXS images for MO cubic phase doped with varying wt%Fc12 .... S3

ESI 3. SAXS peaks indexing for MO cubic phases doped with varying wt%Fc12 .... S4

ESI 4. Stability test on repeated redox cycling of 0.2%Fc12/MO modified electrode........... S6

ESI 5. Long term stability test of Fc12/MO/GOx bioelectrodes S6

ESI 1. Capillary sample preparation protocol for SAXS analysis

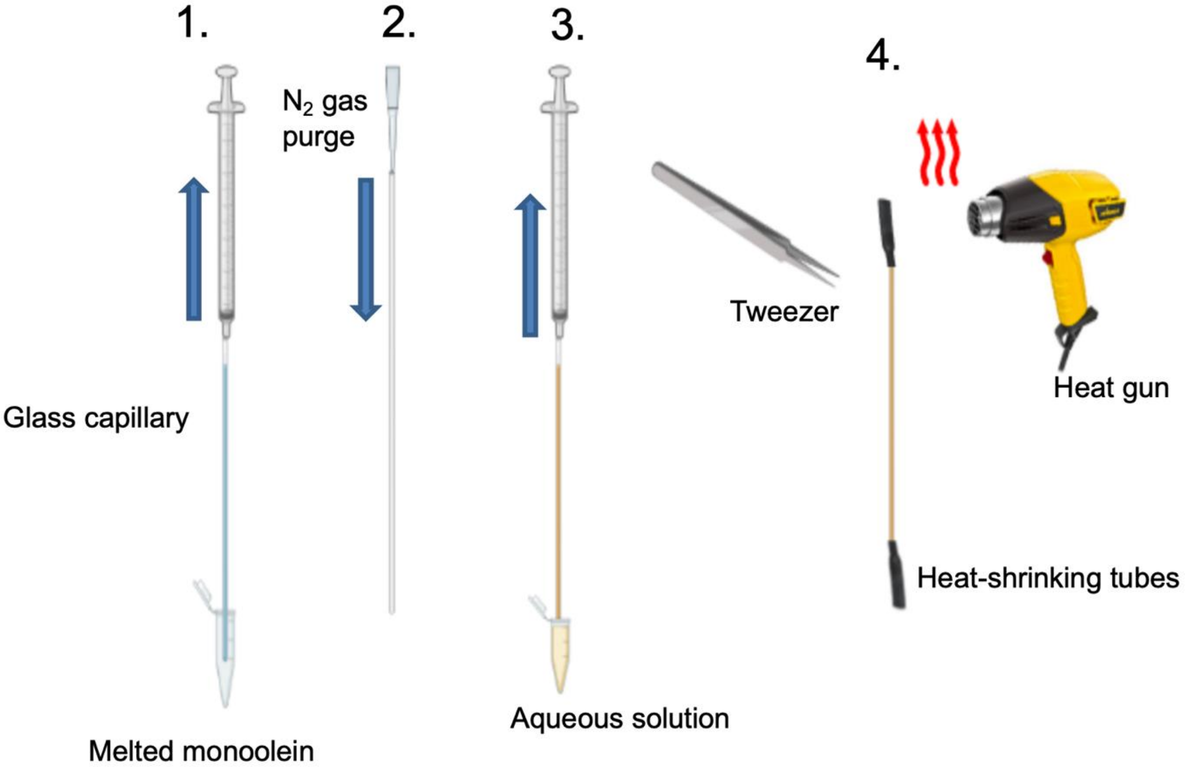

ESI Figure 1. Preparation of capillary samples for SAXS analysis: 1) A glass capillary (d=1.5 mm) was connected to a 1mL syringe using a silicone tubing. Melted monoolein in the Eppendorf was drawn up and fed into the capillary; 2) Excess monoolein was ejected out of the capillary by purging N2 gas at one end to create a layer of lipid coating; 3) The capillary was wiped with EtOH immersed tissues, connected to the syringe, and placed into an Eppendorf contains aqueous solution (buffer or enzyme buffer). The aqueous solution was drawn up using the syringe to fill the capillary. The lipid coating was subsequently exposed to an excess aqueous condition, and the lipid cubic phase was formed inside the capillary; 4) The two ends of the capillary were sealed via two heat-shrinking tubes. The tubes were heated under a heat gun and suppressed with a tweezer to ensure better sealing.

ESI 2. 2D SAXS images for MO cubic phase doped with varying wt%Fc12

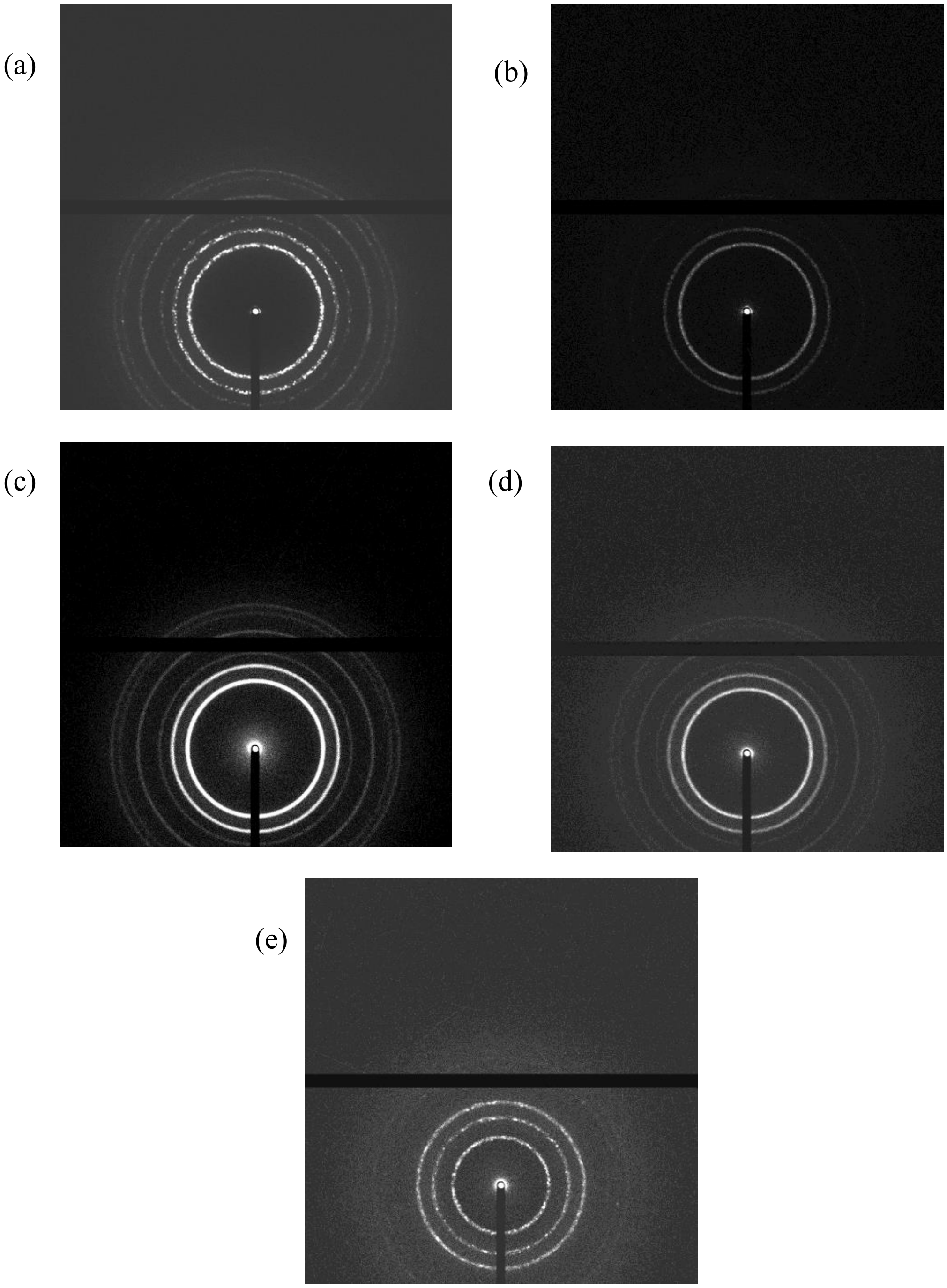

ESI Figure 2. The 2D SAXS patterns collected for different monoolein cubic phases with varying Fc12/MO (w%) compositions at excess aqueous condition: (a) 0%Fc12/MO, (b) 0.2%Fc12/MO, (c) 5%Fc12/MO, (d) 10%Fc12/MO and (e) 15%Fc12/MO

ESI 3. SAXS peaks indexing for MO cubic phases doped with varying wt%Fc12

The phase identity of a MO polymorph or other lyotropic phases can be identified using small-angle X-ray scattering (SAXS). The technique follows similar principles to X-ray powder diffraction (XRPD). A stream of collimated X-ray beams hit the sample of interest at long sample-detector-distance (>300 mm) to provide 2D scattering patterns at small scattering angles, approximately at the range of 0 to 10˚. [1] The scattering patterns correlate to the shape and form features of the sample. After 2D patterns collection, integration is performed to transform data into 1D pattern where one can interpret the Braggs peaks positions at different q positions [2]

$$q = \frac{4\pi}{\lambda} sin\,\theta \quad (1)$$

Where q is the length of the wave scattering vector (nm^{-1}), λ and $sin\,\theta$ adopt the original meaning in the Bragg's law. The relationship between q and the d-spacing, d is as followed [2]

$$d = \frac{2\pi}{q} \quad (2)$$

The lattice parameter *a* (nm) of a cubic phase lattice can be calculated as

$$a = \frac{1}{d} \cdot \sqrt{h^2 + k^2 + l^2} \quad (3)$$

Therefore, the Braggs peaks collected from the 2D-SAXS can be indexed by substituting (2) into (3)

$$a = \frac{q}{2\pi} \cdot \sqrt{h^2 + k^2 + l^2} \quad (4)$$

Where h, k and l are miller indices of the Braggs' peaks . Each type of LCP has its characteristic Braggs reflection patterns and values of $\sqrt{h^2 + k^2 + l^2}$, from which we can solve the phase identity. For example, the cubic phase adopts Pn3m space group has $\sqrt{h^2 + k^2 + l^2}$ ratios as $\sqrt{2}$: $\sqrt{3}$: $\sqrt{4}$: $\sqrt{6}$: $\sqrt{8}$ … .., and Ia3d: $\sqrt{6}$: $\sqrt{8}$: $\sqrt{14}$: $\sqrt{16}$: $\sqrt{20}$….[3]

Bragg peaks indexing for Figure 2 and ESI Figure 2 are displayed in ESI Table 1

ESI Table 1. Space group and lattice parameter indexing for MO cubic phases doped with varying wt%Fc12

Sample	*q* (nm)	hkl	a (nm)	Space Group
0%Fc12	0.95	110	9.35	*Pn3m*
	1.16	111	9.38	
	1.34	200	9.38	
	1.63	211	9.44	
0.2%Fc12	0.95	110	9.35	*Pn3m*
	1.16	111	9.38	
	1.34	200	9.38	
	1.63	211	9.44	
5%Fc12	0.96	110	9.25	*Pn3m*
	1.16	111	9.30	
	1.36	200	9.24	
	1.66	211	9.27	
10%Fc12	0.90	110	9.87	*Pn3m*
	1.09	111	9.84	
	1.28	200	9.81	
	1.57	211	9.80	
15%Fc12	0.67	110	13.26	*Im3m*
	0.96	200	13.09	
	1.17	211	13.15	

Reference

1. Anderson, D. M.; Gruner, S. M.; Leibler, S.; Geometrical aspects of the frustration in the cubic phases of lyotropic liquid crystals. *Proc. Natl. Acad. Sic. USA*. **1988**, *85*, 5364-5368
2. Li, J.; Jiao, A.; Chen, S.; Wu, Z.; Xu, E.; Jin, X.; RETRACTED: Application of the small-angle X-ray scattering technique for structural analysis studies: A review. *J. Mol. Struct*, **2018**, *5*, 391-400
3. Kulkarni, C. V.; Wachter, W.; Iglesias-Salto, G.; Engelskirchen, S.; Ahualli, S.; Monoolein: A magic lipid. *Phys. Chem. Chem. Phys.*, **2011**, *13*, 3004-3021

ESI 4. Stability test on repeated redox cycling of 0.2%Fc12/MO modified electrode

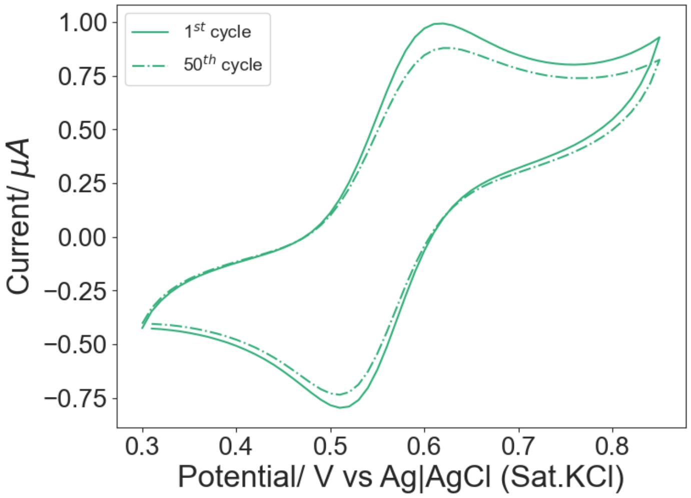

ESI Figure 3. The 1st and 50th cyclic voltammograms of 0.2%Fc12/MO modified electrode collected at 0.1 V s^{-1} in PB solution (pH=7.0) after continuous sweeping potential between 0.3V and 0.85V for 50 cycles

ESI 5. Long term stability test of Fc12/MO/GOx bioelectrodes

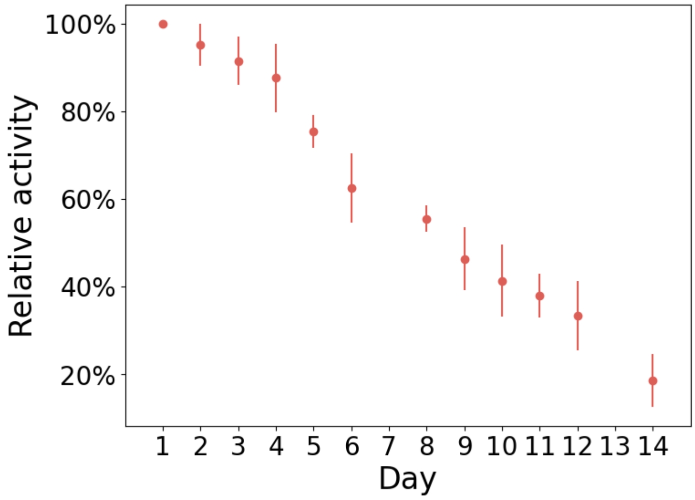

ESI Figure 4. Relative activity of Fc12/MO/GOx over 14 days. The relative activity was calculated by collecting the mass transport limiting current of the bioelectrode in a 6mM glucose PB solution (pH=7.0) on each day and comparing with the results obtained on day 1. (n=3)
